# A Study on the Phytotoxic Potential of the Seasoning Herb Marjoram (*Origanum majorana* L.) Leaves

**DOI:** 10.3390/molecules26113356

**Published:** 2021-06-02

**Authors:** Antonio Cala, José R. Salcedo, Ascensión Torres, Rosa M. Varela, José M. G. Molinillo, Francisco A. Macías

**Affiliations:** Allelopathy Group, Department of Organic Chemistry, School of Science, Institute of Biomolecules (INBIO), University of Cadiz, C/República Saharaui 7, 11510 Puerto Real, Cadiz, Spain; antonio.cala@uca.es (A.C.); jose.salcesempe@alum.uca.es (J.R.S.); chema.gonzalez@uca.es (J.M.G.M.); famacias@uca.es (F.A.M.)

**Keywords:** marjoram, *Origanum majorana* L., bioguided fractionation, wheat coleoptile, bioassay, spice, phytotoxic, flavonoid, monoterpene, diterpene

## Abstract

In the search of new alternatives for weed control, spices appear as an option with great potential. They are rich in bioactive natural products and edible, which might minimize toxicity hazard. Marjoram (*Origanum majorana* L.) is an aromatic herb that has been widely employed as a seasoning herb in Mediterranean countries. Although marjoram boasts a plethora of therapeutic properties (painkiller, antibiotic, treatment for intestinal disorders, etc.), the potential for its extracts for weed control is still to be more thoroughly explored. In order to determine their phytotoxic potential, marjoram leaves were subjected to different bioguided extraction processes, using water, ethyl acetate, acetone or methanol. The most active extract (acetone) was sequentially fractionated to identify its most active compounds. This fractionation led to the isolation and identification of 25 compounds that were classified as monoterpenes, diterpenes or flavonoids. Among them, a new compound named majoradiol and several compounds are described in marjoram for the first time. The phytotoxicity of the major compounds to etiolated wheat coleoptiles was compared against that of the commercial herbicide (Logran^®^), with similar or higher activity in some cases. These results confirm the extraordinary potential of the extracts from this edible plant to develop safer and more environmentally friendly herbicides.

## 1. Introduction

Marjoram (*Origanum majorana* L.) is an aromatic herb that has been grown in several Mediterranean countries since ancient times and whose use became popular during the Middle Ages (around the 16th century) both as a medicinal plant and a seasoning ingredient [1]. Because of its interesting properties, it is presently used in ethnopharmacology in Morocco and Turkey for the treatment of digestive disorders, bug bites or as a disinfectant, among other therapeutic uses [2,3].

The extracts from this edible plant are well known for their prominent and varied biological activities. Apart from their rich aromas and flavours, which determine their culinary value, many other properties, such as anti-anxiety, anticonvulsant, antidiabetic, anti-gout, anti-mutagenic, antiulcer, antibacterial, antifungal, anti-protozoal, insecticidal and ovicidal, have been described [4]. Marjoram extracts have been proposed as preservatives for sausages or ham [5,6] because of their antimicrobial activity, which particularly affects *Blastocystis* spp. [7], *Escherichia coli*, *Aspergillus niger*, *Trichoderma viride* and *Penicillium cyclopium* [8]. Sedative effects on rats have been observed in terpenoid-rich extracts, which are comparable to those of Diazepam^®^ [9]. Phenolic-rich extracts, characterized by their antioxidant properties, have also been obtained [10].

**Figure 1:**
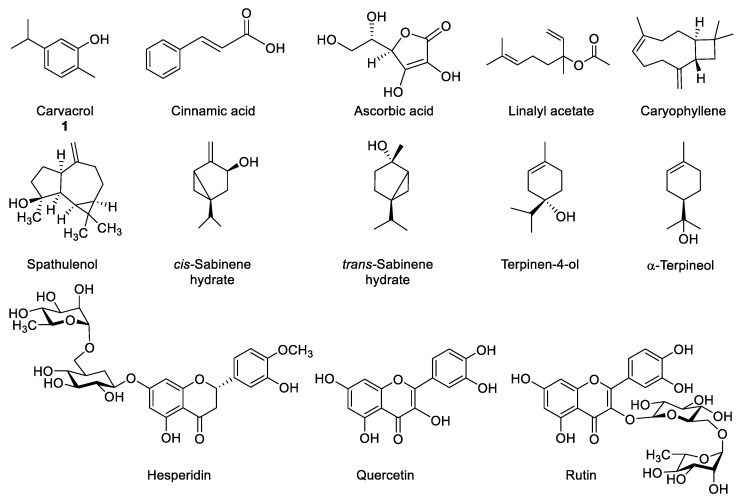
Bioactive components in marjoram (*Origanum majorana*) extracts.

Up to date, many bioactive marjoram constituents have been described, such as: carvacrol, cinnamic acid, ascorbic acid, linalyl acetate, caryophyllene, spathulenol, *cis-* and *trans*-sabinene hydrate, terpinen-4-ol, α-terpineol, hesperidin, quercetin, rutin, etc. (Figure 1). Depending on the origin of each plant, its chemical composition may vary according to the three main chemotypes that have been described until now. Thus, the type that is found in Reunion Island, Greek and Egypt contains mainly terpinen-4-ol and sabinenes [5,11,12], while Turkish marjoram contains mainly carvacrol [13,14] and the Iranian type presents a large content of linalyl acetate [15].

The current demand for safer and environmentally friendly agrochemical products, as well as the increasing concern regarding the resistance to classic herbicides, have motivated different researchers to investigate allelopathic compounds [16]. The use of extracts, enriched in phytotoxic natural products, appears as an attractive alternative for weed control on account of its interesting advantages. Thus, not only complex separation processes are no longer required, but the synergistic effects that arise from allelochemical combination enhance the effectiveness of these compounds on their own [17,18].

Even though, there are numerous reports on the constituents of marjoram essential oils and their bioactivity, scarce information can be found on the phytotoxic activity and agriculture potential of the extracts from this aromatic plant. It has nevertheless been reported that marjoram essential oils have some preservative effect on post harvested fruits against grey and blue mould (*Botrytis cinerea* and *Penicillium expansum*) as well as against Monilinia brown rots [19,20]. Essential oils have also been found to significantly inhibit hypocotyl growth of *P. oleracea* and *L. multiflorum*, as well as both hypocotyls and radicle of *E. crus-galli* [21], although germination was not affected. To our knowledge, although a number of phytotoxicity bioassays have been performed on the essential oils of some related species such as *Origanum vulgare* [22], no further reports on the phytotoxicity of either marjoram essential oils, nor on their extracts are available at present.

**Figure 2:**
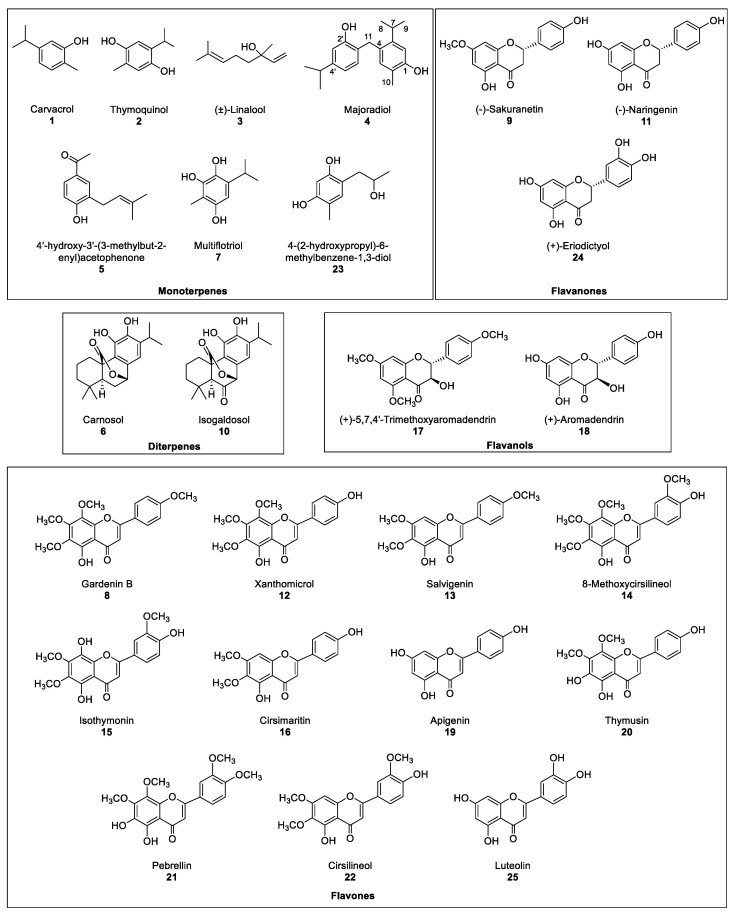
Compounds isolated from the most active fraction in marjoram leave acetone extract.

Therefore, the aim of this work is the evaluation the phytotoxic potential of marjoram leaf extracts and their compounds. Thus, the production of extracts from marjoram leaves and their bioguided fractionation followed by their isolation and structural determination yielded 25 compounds in the most active fraction (Figure 2). Some of those compounds are reported in marjoram for the first time. The whole list includes monoterpenes (**1**–**3**, **5**, **7** and **23**), diterpenes (**6** and **10**) and flavonoids (**9**, **11**–**22**, **24** and **25**). A new compound named majoradiol, a carvacrol dimer (**4**), was found among the first group. The isolated compounds **6**, **8**, **9**, **11**, **12**, **15**, **16**, **18**, **19**, **20**, **24** and **25** were chosen for an etiolated wheat coleoptile bioassay and inhibition of growth was found in all the cases. Phytotoxic activity close or even higher than that of the commercial herbicide Logran^®^ was registered in **9**, **15** and **16**, which would confirm the potential of marjoram extracts as a source of safer and environmentally friendly agrochemicals.

## 2. Results and Discussion

As previously mentioned, even though it is well known that marjoram possesses a wide variety of biological activities, little attention has been paid to its potential as a source of phytotoxic extracts that could be used as an alternative to pesticides for weed control. The extraction method was selected so that the most bioactive marjoram extracts against etiolated wheat coleoptiles would be obtained.

The etiolated wheat coleoptile bioassay was chosen as the method to evaluate the phytotoxic activity of extracts, fractions and products, since it is a sensitive, simple and rapid tool that can be conducted with just a small amount of sample. The undifferentiated meristematic plants cells that can be found in coleoptiles make of them a highly sensitive cellular model that allows evaluation of the effect that extracts, fractions or products have on them. Such effects can be observed macroscopically as the result of the stimulation or inhibition of the coleoptile growth with respect to the negative control. When growth inhibition occurred, the fraction or the product being evaluated was considered as phytotoxic. When no inhibition was registered, the evaluated sample was discarded for any further tests or studies. If the first evaluation was conducted on a particular seed, we could only determine the toxicity of the substance to that particular seed. Consequently, we employed this bioassay as an evaluation tool with a wide scope [23,24].

**Figure 3:**
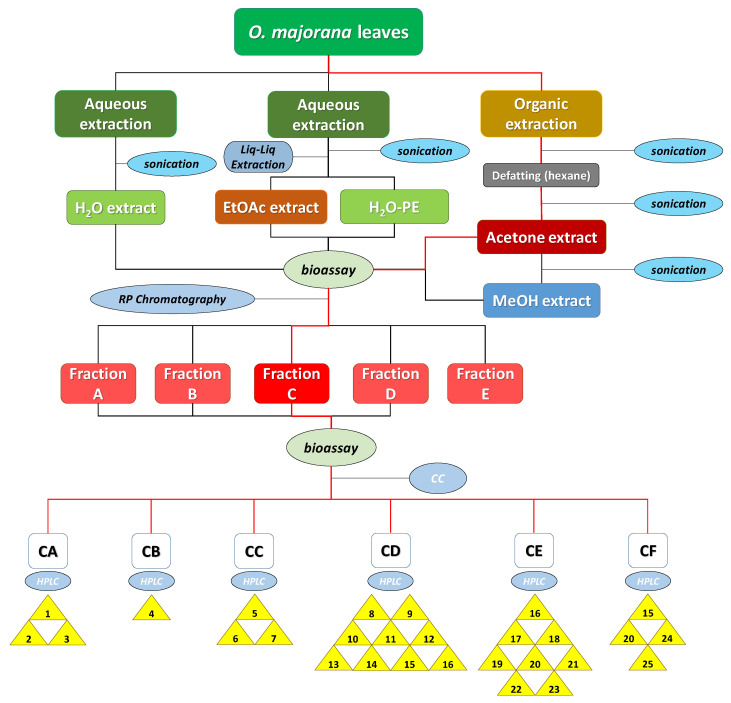
Diagram procedure of the bioguided isolation of *O. majorana* leaves. The red lines indicate the itinerary for the isolation of compounds **1**–**25**.

In a different order of things, the optimal extraction procedure that would result in the highest possible bioactivity levels was to be selected. For this purpose, leaves of marjoram (*O. majorana* L.) were extracted by water and by means of organic solvents following the procedure described in Section 3.3 (summarized in Figure 3). In every case, ground leaves were sonicated for 20 min after adding each of the solvents to increase extraction efficiency.

**Figure 4:**
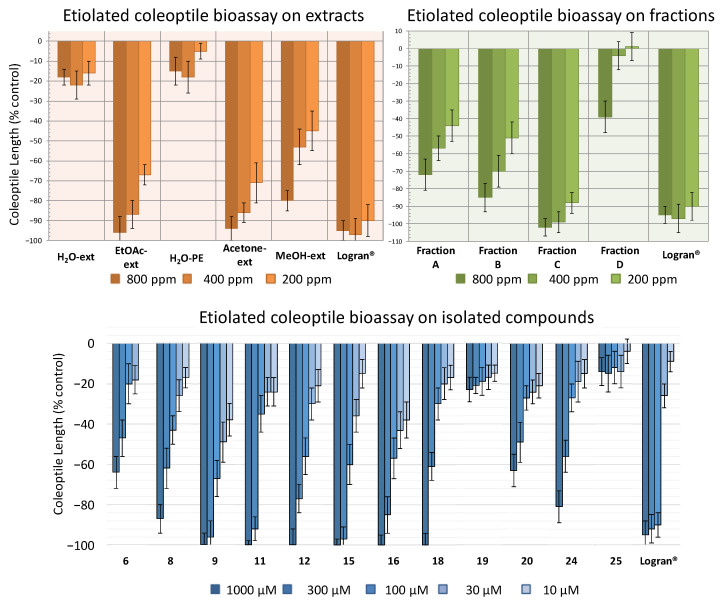
Activity shown by the extracts H_2_O-ext, H_2_O-PE, EtOAc-ext, acetone-ext and MeOH-ext (800–200 ppm), the fractions **A**–**D** (800–200 ppm) and the compounds **6**, **8**, **9**, **11**, **12**, **15**, **16**, **18**–**20**, **24** and **25** (1000–10 μM) according to the bioassay on etiolated wheat coleoptiles. The results are given in % in relation to the control coleoptile length. Positive values indicate a greater growth than the control sample and negative values a lesser growth than the control sample.

Following the procedure described above, five extracts were obtained: aqueous extract (H_2_O-ext), aqueous extract after extraction with EtOAc (H_2_O-PE), organic EtOAc obtained from aqueous extract (EtOAc-ext), and the organic extracts after defatting with hexane, namely the acetone extract (acetone-ext) and methanolic extract (MeOH-ext). The extracts were tested on etiolated wheat coleoptiles (Figure 4), observing the highest inhibitory activities by EtOAc-ext and acetone-ext. The inhibitory activity was significant even at the lowest concentration and was even comparable to that of the commercial herbicide Logran^®^. Both extracts presented similar inhibitory capacity at the tested concentrations, although acetone-ext presented a slightly higher activity at its lowest concentration (200 ppm) as well as higher yields from the extraction process (2.81% instead of 1.89%). Consequently, it was selected as the best extraction method.

The acetone-ext method yielded 9.602 g of extract from 450 g of ground leaves. The main phytochemicals were fractionated and the chlorophyll was removed by reverse phase column chromatography. Thus, fractions **A**–**E**, were obtained, among which fraction **E** contained mainly chlorophyll and was discarded for the subsequent bioassay (Figure 4). In this case, the disparities between the bioactivity levels of the different fractions were significant, with only fraction **C** exhibiting a comparable activity to that of the commercial herbicide Logran^®^, being highly inhibitory at all the tested concentrations (800–200 ppm). Fractions **A** and **B**, on the other hand, presented moderate inhibitory activities, while fraction **D** only achieved a growth inhibitory capacity of around 40%, and only at its highest concentration (800 ppm).

The most active fraction, Fraction **C,** was refractionated using Si-gel to obtain fractions **CA**–**CF**. However, each one of those fractions contained mixtures of several major and minor compounds that required a further and finer separation, which was achieved by semipreparative HPLC. A total of 24 known compounds (**1**–**3** and **5**–**25**) and a new compound **4** (Figure 2) were isolated in the six fractions. The known compounds were identified by acquiring their ^1^H, ^13^C NMR, MS spectra and specific rotation (α), when needed, and by comparison against their data reported in the literature (Section 3.4). Compounds **1**, **3**, **6**, **9**, **11**, **18**, **19**, **20** and **24** had been previously identified in *Origanum majorana* [14,22,25,26,27]. On the contrary, and to our knowledge, compounds **2**, **5**, **7**, **10**, **15**, **17** and **23** [28,29,30,31,32] were described in this species for the first time, while compounds **8**, **12**–**14**, **16**, **21** and **22** had only been described in other *Origanum* spp. [25]. The compounds could be classified as monoterpenes (**1**–**5**, **7** and **23**), diterpenes (**6** and **10**), flavanones (**9**, **11** and **24**), flavanols (**17** and **18**) and flavones (**8**, **12**–**16**, **19**–**22** and **25**).

Compound **4** was isolated as a yellow oil from fraction **CB** whose molecular formula is C_20_H_26_O_2_, as can be deduced from its HR ESIMS spectrum with m/z calculated for [M-H]^−^ 297.1855 and found 297.1860. The ^1^H NMR spectral data (Section 3.4) showed two groups of signals, three doublets (δ 6.82, 6.70, dd, and 6.66) and two singlets (δ 6.84 and 6.74) at the aromatic hydrogen zone, each integrating for 1H, which is in agreement with the presence of two aromatics rings. The analysis of the coupling constants of the first group of signals agrees with a 1,2,4-substitution (H-6’, δ 6.82, *J* = 7.8 Hz; H-5’, δ 6.70, dd, *J* = 7.8 and 1.7 Hz; and H-3’, δ 6.66, d, *J* = 1.7 Hz). The singlets at δ 6.84 (H-3) and 6.74 (H-6) which belong to the second aromatic ring indicate a tetrasubstitution, with protons in *para* position. Two broad singlets were noted at δ 4.68 and 4.55, each integrating for 1H indicated the presence of two hydroxyl groups, which agrees with the broad band centred at 3130 cm^−1^ that can be observed in the FTIR spectrum and the molecular formula. A broad doublet at δ 3.89 (2H) in the ^1^H NMR spectrum correlated in the HMBC 2D experiment with C-3, and C-6’ as well as with four quaternary carbons (δ 153.6, 146.8, 128.2 and 124.5). Thus, this methylene (H-11) should connect the two aromatic rings. At low field (δ 3.25–1.00), seven signals were observed that indicated the presence of two *iso*propyl groups [δ 3.08 (H-7, sp, *J* = 7.0 Hz, 1H), δ 1.12 (H-8, H-9, d, *J* = 7.0 Hz, 6H); and 2.83 (H-7’, sp, *J* = 6.7 Hz, 1H), δ 1.21 (H-8’, H-9’, d, *J* = 6.7 Hz, 6H)] that correlated in the ^1^H-^1^H-COSY experiment. A singlet (3H) at δ 2.16 (H-10) was assigned to a methyl group that correlated with a carbon at δ 15.4 (C-10) in the HSQC. By including the expected two hydroxyls, the two isopropyl groups and the methyl group, the substitution pattern of the two-ring system was found to be similar to that of a dimer of carvacrol (**1**). The position of each substituent in each aromatic ring, as well as the assignment of each quaternary carbon, was confirmed by the HSQC and HMBC experiments. (Information on compound **4** is showed in the Supplementary Material).

Therefore, the structure of compound **4** was determined to be 4-(2-hydroxy-4-isopropylbenzyl)-5-*iso*propyl-2-methylphenol, which was described for the first time and given the name of majoradiol.

In order to determine the candidates responsible for the high activity of fraction **C**, the major compounds isolated in the HPLC were tested. Only compounds **6**, **8**, **9**, **11**, **12**, **15**, **16**, **18**–**20**, **24** and **25**, were chosen for the bioassay on etiolated wheat coleoptiles, using the range of 1000–10 μM (Figure 4). The criteria for the selection of those compounds were the amount obtained and whether information was available regarding their phytotoxicity in the literature. Monoterpenes, such as **1** and **3**, were already described as phytotoxic compounds that can be found in the essential oils of several species [33]. Regarding the new compound **4**, only 2.3 mg had been obtained, which was not enough to carry out the bioassay.

All the tested compounds inhibited coleoptile growth, with flavanone **9** standing out as the most active compound at every concentration, even surpassing the commercial herbicide at the lowest concentration. It is also worth mentioning the flavones **15** and **16** had similar activity to **9**, although **15** had lesser activity than the other two at the lowest concentration tested. On the other hand, diterpene **6** and flavone **20** only presented a moderate inhibitory activity while flavones **19** and **25** exhibited low levels of inhibitory capacity, even at the highest concentration.

The bioactivity data were treated statistically to determine their IC_50_ values, and clog*P* values were calculated (Table 1). The clog*P* values varied between 1.368 and 3.163 and the molecular weight of the tested compounds were all in the range 160–500 uma, following in both cases Lipinski’s rule of five [34,35]. However, a clear correlation with cLog*P* was not found, since compounds with opposite activities presented similar values, such as the highly active flavanone **9** (IC_50_ 32.3 μM, clog*P* 2.967) and the lesser active flavone **19** (IC_50_ > 1000 μM, clog*P* 2.905). Nevertheless, some structure-activity relationships (SAR) could be discussed. Most of the flavonoids (**8**, **9**, **11**, **12**, **15**, **16**, **18**, **24**) were more active than the diterpene **6**, and among the flavonoids, the methylated compounds were more active than their non-methylated counterparts. Hence, flavanone **9** (IC_50_ 32.3 μM) was more active than flavanones **11** (IC_50_ 123 μM) and **24** (IC_50_ 275 μM); while flavones **8** (IC_50_ 159 μM), **12** (IC_50_ 84.5 μM), **15** (IC_50_ 58.3 μM), **16** (IC_50_ 56.6 μM) and **20** (IC_50_ 496 μM) were more active than **19** and **25** (IC_50_ > 1000 μM on both). In addition, the absence of the double bond at C_2_-C_3_ on flavanones **9**, **11** and **24** as well as on flavanol **18** had a beneficial effect on their activity levels when compared against the flavones.

The most active compound sakuranetin (**9**), was reported previously as a compound with anti-inflammatory activity on COX-1, similarly to naringenin (**11**), aromadendrin (**18**) or eriodictyol (**24**) [36]. Carnosol (**6**), with a moderate activity, had been previously found to exhibit antioxidant activity [37], which is expected from abietane diterpenoids that have been associated with a wide spectrum of biological activities, such as anti-inflammatory, antimalarial, cytotoxic, antimicrobial, etc [38]. On the other hand, the least inhibitory compounds, apigenin (**19**) and luteolin (**25**), are also well-known natural products with other biological activities such as antioxidant [37] or antibacterial [39]. The wide array of biological activities exhibited by these compounds corroborate the ample range of therapeutic properties that have been attributed to marjoram.

The large number of bioactive compounds isolated from fraction **C**, enriched with the major active compounds from the acetone-ext, allowed fraction **C**, by itself, to inhibit coleoptile growth by almost 90% at the lowest tested concentration of 200 ppm. As previously mentioned, different extracts from marjoram have already been used for its therapeutic properties, apart from the gastronomic use that it is given in certain Mediterranean countries. In addition, some of the compounds isolated from the extracts have already been confirmed to present phytotoxic properties against certain weeds. Thus, carvacrol (**1**) is phytotoxic against several *Amaranthus* spp. [34]. The increasing restrictions on the use of agrochemicals in crops, i.e., the potential ban on glyphosate in the near future [40,41], triggers the need to develop alternative methods for weed control. The use of natural product enriched extracts such as those obtained from marjoram, might be a good alternative as a pre-emergence herbicide, particularly for low-resource countries. In addition, marjoram is a seasoning herb that has been long used for cooking and human consumption and, therefore, a reduced toxicity is to be expected from its extracts in comparison to other phytochemical products.

## 3. Materials and Methods

### 3.1. General Experimental Procedures

The level of purity of the compounds was determined by ^1^H NMR spectroscopy and every compound was purified in the HPLC prior to the bioassay. ^1^H and ^13^C NMR spectra were recorded at 400 MHz, 500 MHz and 600 MHz by means of Agilent spectrometers (Palo Alto, CA, USA), equipped with a Z-gradient module and a 5 mm Oneprobe for liquids with auto-tuning. The COSY-45, HSQC and HMBC experiments were performed using Varian vnmrj microprograms. Either CDCl_3_ or CD_3_OD (MagniSolv™, Merck, Darmstadt, Germany) were used to dissolve the samples. The residual peak of the solvent was used as internal standard in each case. The mass spectra were recorded in the negative-ion mode in the range m/z 100-2000, with a mass resolution of 20,000 and an acceleration voltage of 0.7 kV on a UPLC-QTOF ESI (Waters Synapt G2, Manchester, UK) high resolution spectrometer. The FTIR spectra were obtained by means of a Perkin-Elmer Spectrum TWO IR spectrometer. The major absorptions in the infrared are given as wavenumbers (ῦ) in cm^−1^. The optical rotations were measured in CHCl_3_ on a JASCO (Tokyo, Japan) P-2000 polarimeter. TLC were performed on silica gel (Merck, Darmstadt, Germany) Kieselgel 60, F_254_ and RP-18 F_254_S aluminium sheets. The spots were visualized by exposure to UV radiation, or by spraying with H_2_O/AcOH/H_2_SO_4_ (20:4:1) solution, followed by the application of a heating-gun. The chromatographic columns (CC) were performed using Kieselgel 60 silica gel (Merck), 0.063–0.200 mm; and LiChroprep RP-18, 0.040–0.063 mm. The HPLC separation in the isocratic mode, with flow 3 mL·min^−1^, was performed using a Merck Hitachi D-7000 equipped with a RI detector and a 200 μL injector. A semipreparative Phenomenex (Torrance, CA, USA) Luna 250 mm × 10 mm Si 100 Å (10 μm) column equipped with a Si Security Guard SemiPrep Cartridge 10 mm × 10 mm was chosen for the purifications. HPLC grade solvents were employed for all the purifications. Either Sigma-Aldrich Co. (St. Louis, MO, USA), Merck or Alfa Aesar (Ward Hill, MA, USA) supplied the reagents and the solvents. The seeds for the etiolated wheat coleoptile bioassay were kindly supplied by Fitó (Barcelona, Spain).

### 3.2. Plant Material

Dried leaves of marjoram (*Origanum majorana* L.) were purchased from an aromatic herbs retailer in Granada (Spain). A sample of the original plant material is stored in the laboratory of Allelopathy in the Department of Organic Chemistry (University of Cadiz).

### 3.3. Bioguided Extraction and Purification of Natural Products from O. majorana *L.* leaves

In order to optimize the procedure for the extraction of the bioactive metabolites in *O. majorana* leaves, 10 g of ground leaves were subjected to extraction and then used for etiolated wheat coleoptile bioassay. Their activity levels were then measured to identify the most active extracts.

First of all, 10 g of ground leaves were added 100 mL of deionised H_2_O and sonicated for 20 min. The supernatant was filtered off and the extraction of the solid residue was repeated for a total of three times. The combined three supernatants were distilled by means of a rotatory evaporator and 1.458 g of solid residue (14.58% yield, H_2_O extract, H_2_O-ext) were obtained. This extraction procedure was repeated a second time with another 10 g of leaves but, this time, the combined supernatants were extracted using 100 mL of EtOAc × 3. The organic layers were combined and dried over anhydrous Na_2_SO_4_ and then filtered. Both extracts were distilled in a rotatory evaporator and 1.178 g of solid residue from the aqueous layer (11.78% yield, H_2_O post-extraction extract, H_2_O-PE) as well as 189.7 mg from the organic layer (1.89% yield, EtOAc extract, EtOAc-ext) were obtained.

Alternatively, 10 g of ground leaves were extracted directly using organic solvents in a sequential order. Firstly, a defatting process was carried out using 100 mL hexane and sonication for 20 min. The supernatant was filtered off and the resulting solid was defatted two additional times. The defatted residue was added 100 mL acetone to repeat the extraction procedure. The combined acetone supernatants were distilled and 281.3 mg of dry residue (2.81% yield, acetone extract, acetone-ext) were obtained. Lastly, the plant leftover material was extracted for the last time using 100 mL methanol following the same procedure as above described and 487.3 mg of dry residue (4.87% yield, methanol extract, MeOH-ext) were obtained.

**Figure 5:**
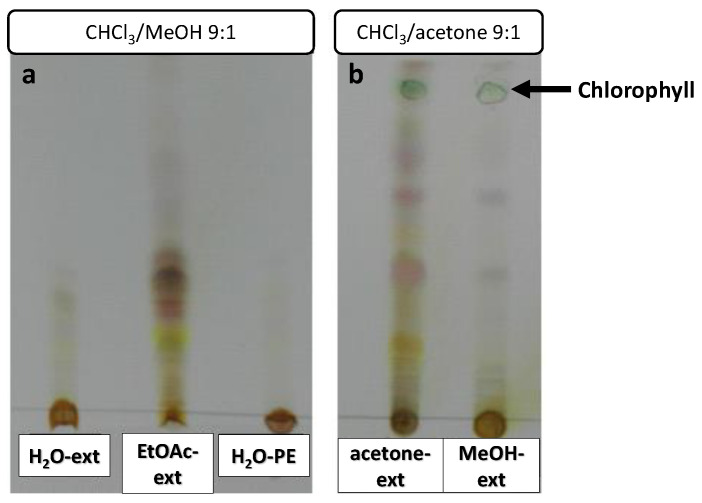
TLCs of *O. majorana* extracts, eluted with CHCl_3_/MeOH 9:1 (**a**) and CHCl_3_/acetone 9:1 (**b**).

Extract samples were subjected to TLC to confirm the presence of organic compounds (Figure 5). Chlorophyll could be observed as a green spot in the acetone-ext and MeOH-ext TLC (Figure 5b). A number of highly polar compounds were observed in all the extracts, though mid-polarity compounds were only observed in EtOAc-ext and acetone-ext.

In order to select the best extraction method, the bioactivity levels of the 5 extracts obtained were tested on etiolated wheat coleoptiles (Section 3.5). According to the data, EtOAc-ext and acetone-ext were the most active extracts. The latter one was selected because of its larger extraction yields. The previously described acetone extraction procedure (Figure 3) was applied to 450 g of ground leaves and 9.602 g of extract (2.13% yield) were obtained.

A first fractionation was performed to remove the chlorophyll from the acetone-ext by depositing the extract on a 10 cm × 10 cm number 4 glass crucible filter containing 3 cm RP-18. The elution was carried out employing a gradient of MeOH/H_2_O from 1:4 to 1:0, and then flushing it down with CH_2_Cl_2_. 5 groups of fractions were obtained as follows: **A** (314.6 mg, 3.43%), **B** (618.1 mg, 6.43%), **C** (1.182 g, 12.31%), **D** (4.745 g, 49.4%) and **E** (823.0 mg, 8.57%), which had been eluted at 1:4, 2:3, 3:2–4:1, 1:0 and CH_2_Cl_2_, respectively. The activity of fractions **A**–**D** was determined through a new bioassay, while fraction **E,** containing mainly chlorophyll, was discarded.

The most active fraction, fraction **C,** was refractionated by Si-gel CC and eluted using a gradient of Hexane/EtOAc from 9:1 to 0:1, and then flushed down with MeOH, to obtain the following 6 fractions: **CA** (14.7 mg, 1.24%), **CB** (16.8 mg, 1.42%), **CC** (15.3 mg, 1.29%), **CD** (162.4 mg, 13.73%), **CE** (76.4 mg, 6.46%) and **CF** (83.3 mg, 7.52%). Each of these fractions was further purified by semipreparative HPLC in order to isolate the compounds **1**–**25** (Figure 2).

Fraction **CA** was purified by 3 injections eluted with hexane/EtOAc 9:1, to yield 3 major peaks with retention times (t_R_, min.): 12.3 (**1**, 13.8 mg), 14.2 (**2**, 1.9 mg) and 16.5 (**3**, 1.8 mg). Fraction **CB** was purified by 5 injections eluted with hexane/EtOAc 17:3, to yield only 1 significant peak with t_R_ (min.): 18.3 (**4**, 2.3 mg). Fraction **CC** was purified by 5 injections eluted with hexane/EtOAc 7:3, to yield 3 major peaks with t_R_ (min.): 11.5 (**5**, 2.1 mg), 12.7 (**6**, 13.8 mg) and 14.4 (**7**, 2.2 mg). Fraction **CD** was purified by 20 injections eluted with hexane/EtOAc 1:1, to yield 10 peaks with t_R_ (min.): 7.1 (**8**, 9.6 mg), 8.2 (**9**, 2.5 mg), 8.9 (**10**, 2.2 mg), 9.1 (**11**, 2.0 mg), 9.6 (**12**, 4.0 mg), 10.2 (**13**, 3.0 mg), 10.9 (**14**, 1.8 mg), 11.3 (**15**, 2.6 mg) and 12.9 (**16**, 3.6 mg). Fraction **CE** was purified by 10 injections eluted with hexane/EtOAc 2:3, to yield 8 peaks with t_R_ (min.): 5.0 (**16**, 1.1 mg), 6.1 (**17**, 5.0 mg), 6.9 (**18**, 2.6 mg), 7.1 (**19**, 1.8 mg), 8.6 (**20**, 3.8 mg), 9.2 (**21**, 2.5 mg), 10.1 (**22**, 2.9 mg) and 10.7 (**23**, 2.5 mg). Lastly, fraction **CF** was purified by 10 injections eluted with hexane/EtOAc 7:13, to yield 4 major peaks with t_R_ (min.): 8.3 (**15**, 1.4 mg), 9.2 (**20**, 1.3 mg), 10.7 (**24**, 2.1 mg) and 11.3 (**25**, 2.3 mg).

### 3.4. Characterization of the Compounds

Compounds **1**–**3** and **5**–**25**, were identified by comparing their ^1^H and ^13^C NMR, MS spectra and their α value (when required) against certain natural products previously reported in the literature as follows: carvacrol (**1**) [42], thymoquinol (**2**) [28], linalool (**3**) [43], 4’-hydroxy-3’-(3-methylbut-2-enyl)acetophenone (**5**) [44], carnosol (**6**) [45], multiflotriol (**7**) [46], gardenin B (**8**) [47], sakuranetin (**9**) [36], isogaldosol (**10**) [30], naringenin (**11**) [36], xanthomicrol (**12**) [48], salvigenin (**13**) [49], 8-methoxycirsilineol (**14**) [50], isothymonin (**15**) [50], cirsimaritin (**16**) [39], 5,7,4’-trimethoxyaromadendrin (**17**) [31], aromadendrin (**18**) [36], apigenin (**19**) [51], thymusin (**20**) [52], pebrellin (**21**) [53], cirsilineol (**22**) [39], 4-(2-hydroxypropyl)-6-methylbenzene-1,3-diol (**23**) [29], eriodictyol (**24**) [36] and luteolin (**25**) [54].

Compound **4** (Figure 2) is a new compound that has been given the name of majoradiol, a yellow oil with spectroscopic data as follows: HRMS, m/z calcd for C_20_H_25_O_2_ 297.1855, found 297.1860 [M-H]^-^; IR ῦ_max_ 3130 (O-H), 1517, 1493, 1459 (C=C) cm^–1^. ^1^H NMR (500 MHz, CDCl_3_, δ, ppm): 6.84 (s, 1H, H-3), 6.82 (d, *J* = 7.8 Hz, 1H, H-6’), 6.74 (s, 1H, H-6), 6.70 (dd, *J* = 7.8, 1.7 Hz, 1H, H-5’), 6.66 (d, *J* = 1.7 Hz, 1H, H-3’), 4.68 (brs, 1H, OH), 4.55 (brs, 1H, OH), 3.89 (brs, 2H, H-11), 3.08 (sp, *J* = 6.7 Hz, 1H, H-7), 2.83 (sp, *J* = 7.0 Hz, 1H, H-7’), 2.16 (s, 3H, H-10), 1.22 (d, *J* = 7.0 Hz, 6H, H-8’, H-9’), 1.13 (d, *J* = 6.7 Hz, 6H, H-8, H-9). ^13^C NMR (125 MHz, CDCl_3_, δ, ppm): 153.6 (C-2’), 152.9 (C-1), 148.8 (C-4’), 146.8 (C-5), 132.5 (C-3), 130.2 (C-6’), 128.2 (C-4), 124.5 (C-1’), 121.1 (C-2), 119.0 (C-5’), 113.7 (C-3’), 112.5 (C-6), 33.8 (C-7’), 32.2 (C-11), 28.9 (C-7), 24.1 (C-8’, C-9’), 23.9 (C-8, C9), 15.4 (C-10).

### 3.5. Etiolated Wheat Coleoptile Bioassay

The bioactivity of the extracts H_2_O-ext, H_2_O-PE, EtOAc-ext, acetone-ext and MeOH-ext, the fractions **A**–**D**, and the compounds **6**, **8**, **9**, **11**, **12**, **15**, **16**, **18**–**20**, **24** and **25** was determined by bioassay on etiolated wheat coleoptiles. The bioassays were conducted according to the conditions reported in the literature [55], which have been replicated in this study, where the same herbicide (Logran^®^) was used as the positive control and the buffer solution as the negative control. The wheat seeds (*Triticum aestivum*) of the ‘catervo’ variety were kindly provided, free of charge, by ‘Semillas Fitó’ (Spain). All the samples were solved in 0.5% dimethyl sulfoxide and produced clear solutions at all the concentrations tested (800–200 ppm or 1000–10 μM). The results are shown in Figure 4.

### 3.6. Calculation of IC_50_ and clogP Values

The bioactivity data were fitted to a sigmoidal dose-response model using the GraphPad Prism v.5.00 software package [56] to obtain the IC_50_ values that can be seen Table 1. The clog*P* values were obtained by means of the appropriate tool in ChemDraw Professional v.18.0 (PerkinElmer, Waltham, MA, USA) [57].

## 4. Conclusions

One new aromatic terpene, named majoradiol (**4**), as well as **14** known compounds (**2**, **5**, **7, 8**, **10**, **12**–**17** and **21**–**23**) from the terpene and flavonoid families, have been isolated from marjoram leaves (*O. majorana*) for the first time. A total of **12** of the **25** compounds isolated from the most active fraction of a marjoram leaves extract were tested on etiolated wheat coleoptiles. All of them displayed inhibitory activity, which in **9**, **15** and **16** was comparable to that of the commercial herbicide Logran^®^. According to the data obtained from our work, it has been demonstrated that certain culinary spices represent a potential valuable source of phytotoxic compounds, which should be further investigated and developed for their use as natural and environmentally friendly alternative pesticides for weed control. Thus, certain extracts enriched with natural products from Mediterranean cooking spices may embody a suitable alternative for a more integrated and environmentally friendly weed control, especially in low-resource countries where access to modern herbicides may be rather limited.

## Figures and Tables

**Table 1:** IC_50_ and clog*P* values for compounds **6**, **8**, **9**, **11**, **12**, **15**, **16**, **18**–**20**, **24** and **25**. IC_50_ was calculated only for those that reached 50% of inhibitory activity in the bioassay.

Compound	IC_50_ (μM)	R^2^	clog*P*	Compound	IC_50_ (μM)	R^2^	clog*P*
**6**	412	0.979	3.163	**18**	215.4	0.968	1.368
**8**	159	0.986	3.033	**19** ^a^	-	-	2.905
**9**	32.3	0.969	2.967	**20**	496	0.953	2.273
**11**	123	0.937	2.445	**24**	275	0.988	1.848
**12**	84.5	0.988	2.448	**25** ^a^	-	-	2.311
**15**	58.3	0.986	1.871	Logran^®^	42.9	0.979	-
**16**	56.6	0.946	2.860				

^a^ Tested compound below 50% inhibitory activity.

## Supplementary Materials

The following are available online, S2: ^1^H and ^13^C NMR spectra of compound **4**, S3: COSY spectrum of compound **4**, S4: HSQC and HMBC spectra of compound **4**, S5: HRMS spectrum of compound **4**.
