# Supplementary material for: A Study on the Phytotoxic Potential of the Seasoning Herb Marjoram (Origanum majorana L.) Leaves
Source: Molecules. 2021 Jun 2;26(11):3356. doi: 10.3390/molecules26113356 (PMC8199622; doi:10.3390/molecules26113356)
Supplement: Supplementary file 1 [file molecules-26-03356-s001.zip › molecules-1246832-supplementary.pdf]

SUPPORTING INFORMATION

A Study on the Phytotoxic Potential of the Seasoning Herb Marjoram (*Origanum majorana* L.) Leaves

Antonio Cala, José R. Salcedo, Ascensión Torres, Rosa M. Varela, José M. G. Molinillo and Francisco A. Macías*

Allelopathy Group, Department of Organic Chemistry, School of Science, Institute of Biomolecules (INBIO), University of Cádiz, C/ República Saharaui 7, 11510-Puerto Real, Cadiz, Spain; antonio.cala@uca.es (A.C.), jose.salcesempe@alum.uca.es (J.R.S.S.), chon.torres@uca.es (A.T.); rosa.varela@-uca.es (R.M.V.), chema.gonzalez@uca.es, famacias@uca.es (F.A.M.).

*Correspondence: famacias@uca.es

Table of Contents

S2. **^{1}H and ^{13}C NMR spectra of 4**

S3. **COSY spectrum of 4**

S4. **HSQC and HMBC spectra of 4**

S5. **HRMS spectrum of 4**

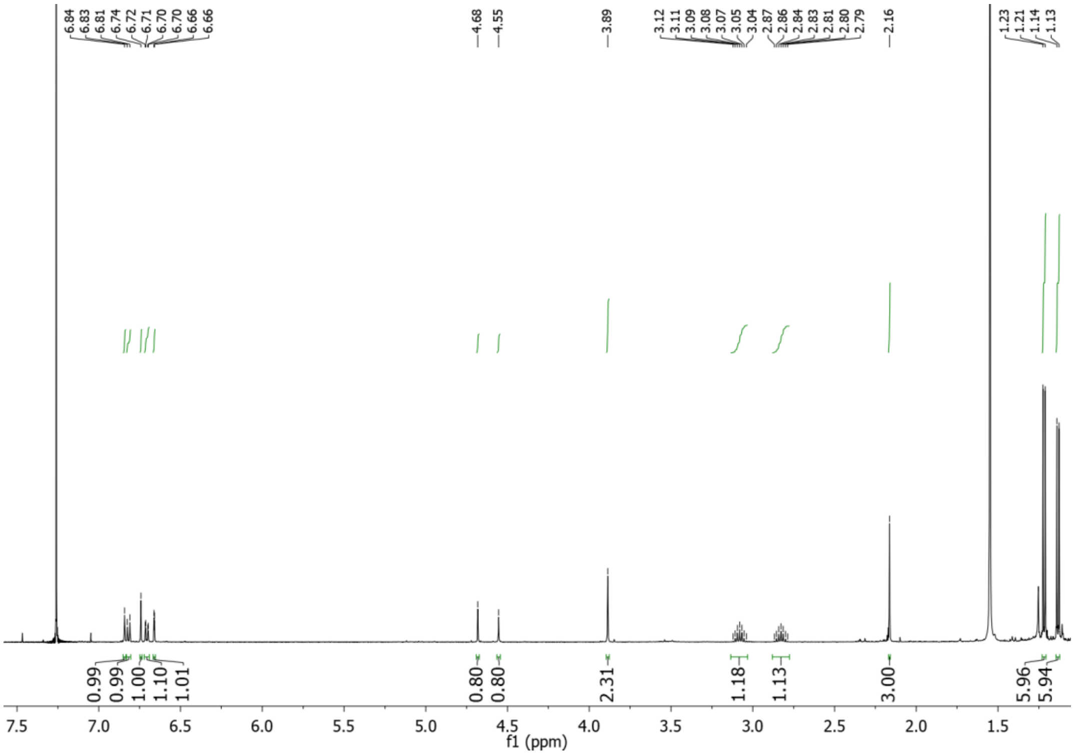

^{1}H-NMR Spectrum of **4** (500MHz, $CDCl_3$)

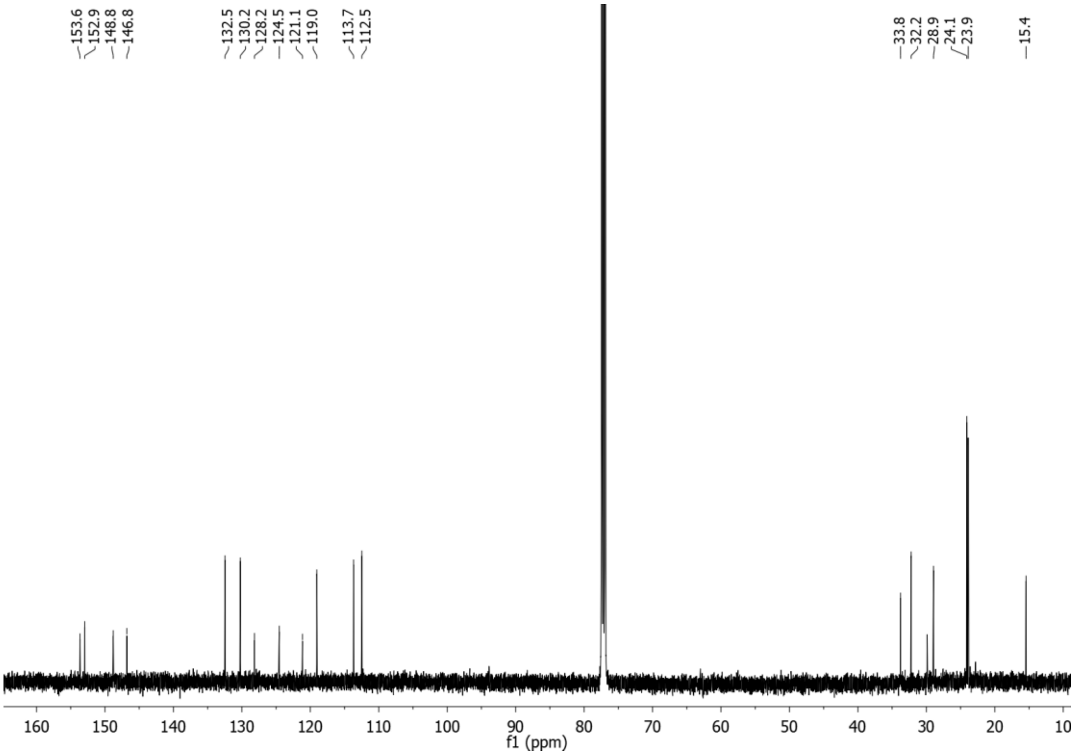

^{13}C-NMR Spectrum of **4** (125 MHz, $CDCl_3$)

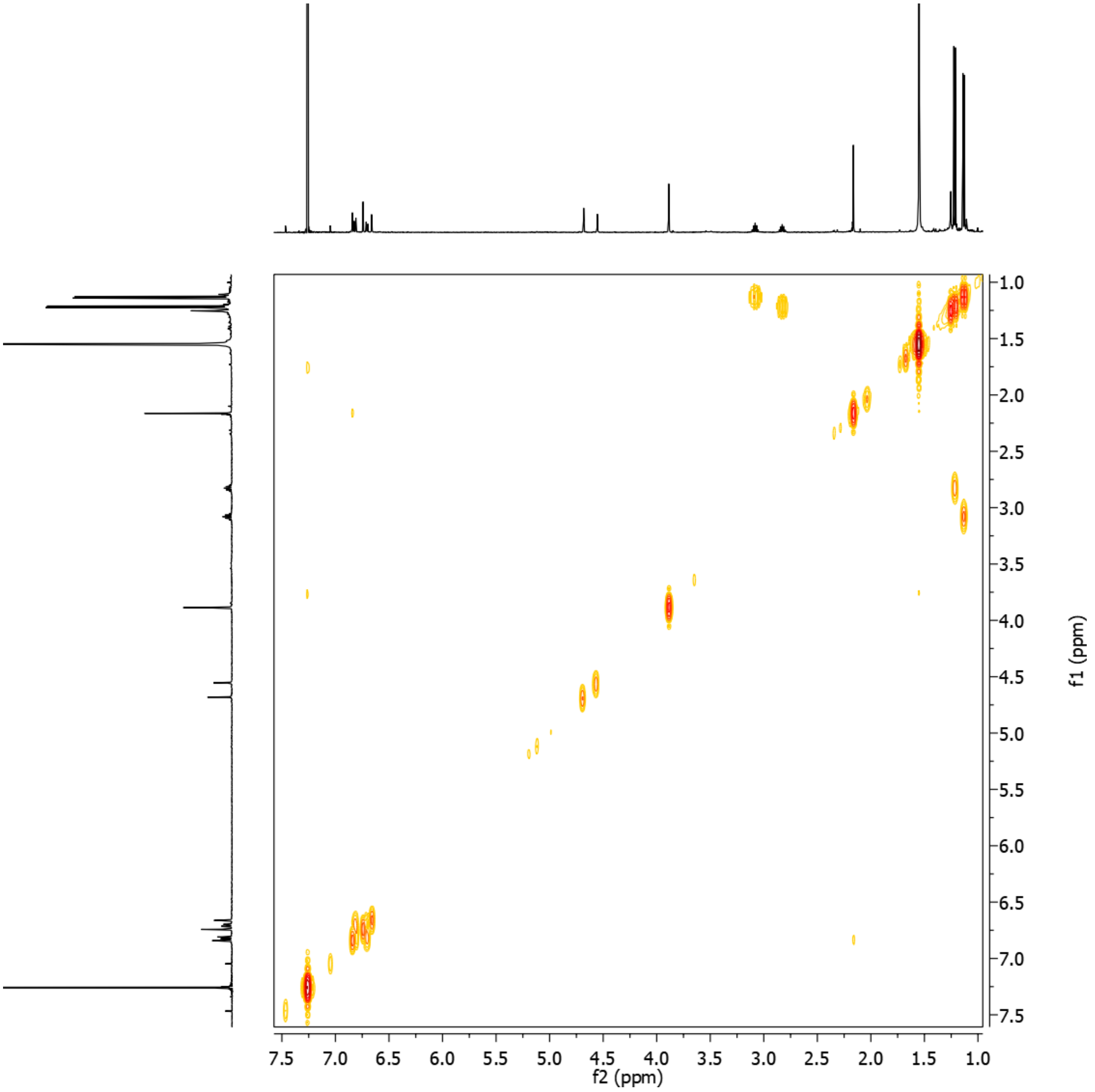

COSY Spectrum of **4** (500MHz, $CDCl_3$)

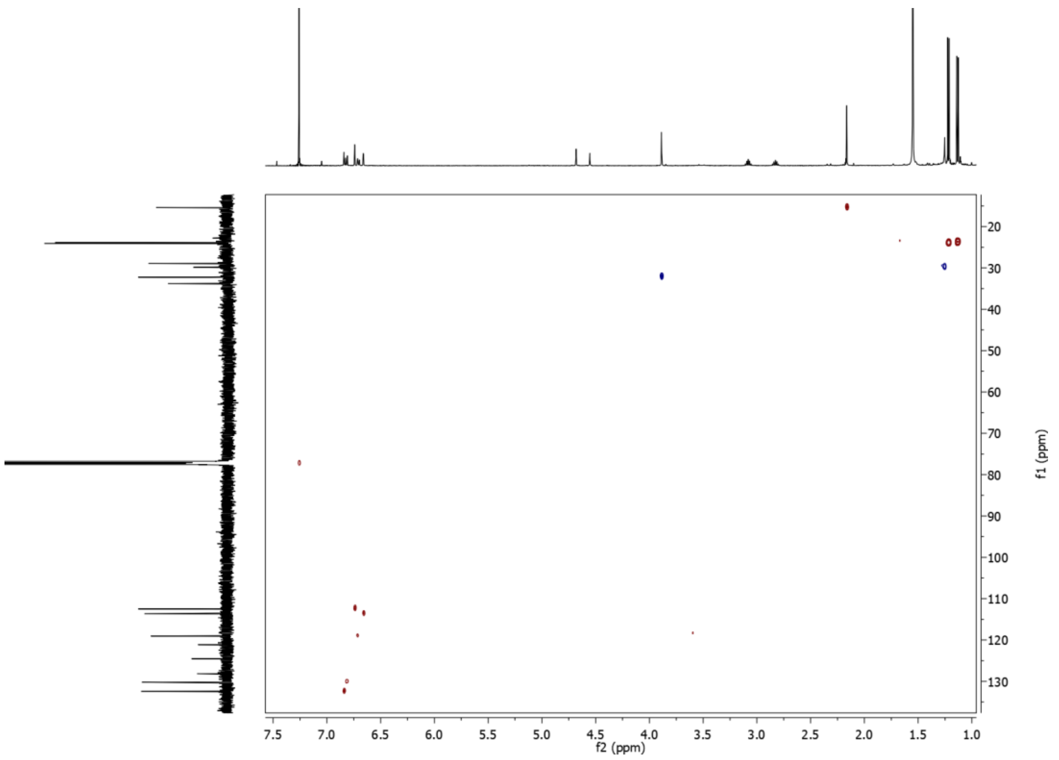

HSQC Spectrum of **4** (500MHz, $CDCl_3$)

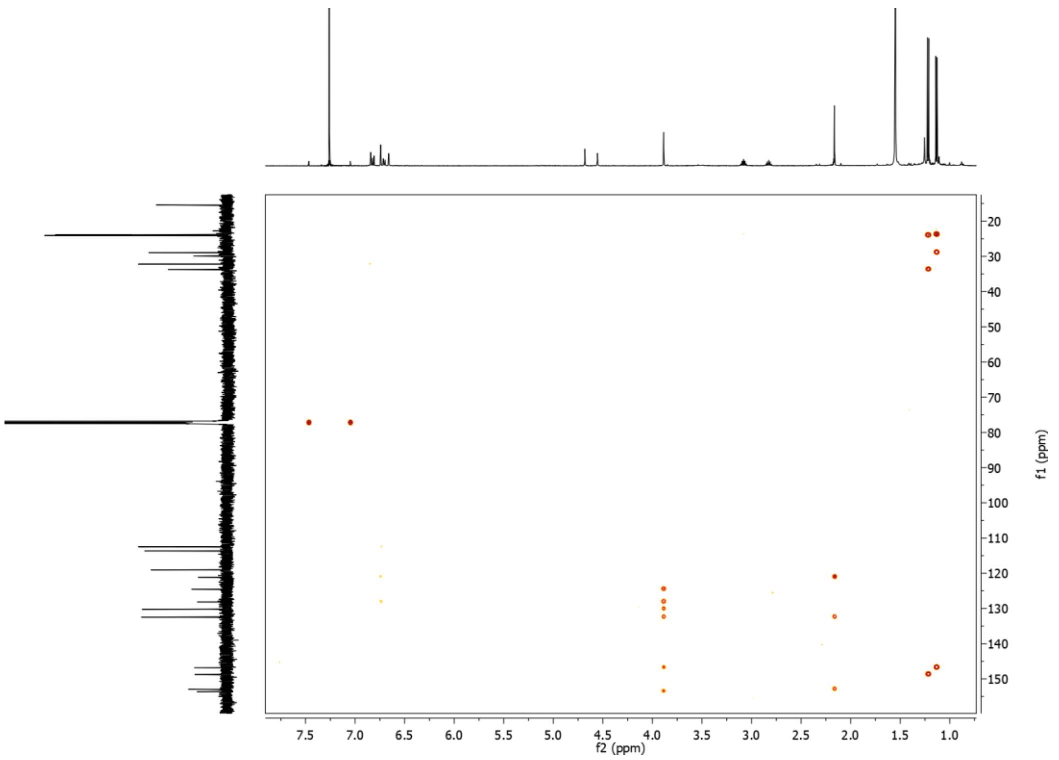

HMBC spectrum of **4** (125 MHz, $CDCl_3$)

Elemental Composition Report

Single Mass Analysis
Tolerance = 2.0 mDa / DBE: min = -1.5, max = 80.0
Element prediction: Off
Number of isotope peaks used for i-FIT = 3

Monoisotopic Mass, Even Electron Ions
96 formula(e) evaluated with 1 results within limits (up to 10 best isotopic matches for each mass)
Elements Used:
C: 0-100 H: 0-100 O: 0-20 Na: 0-1
MEJORADIOL-NEG 489 (4.530)
1: TOF MS ES-

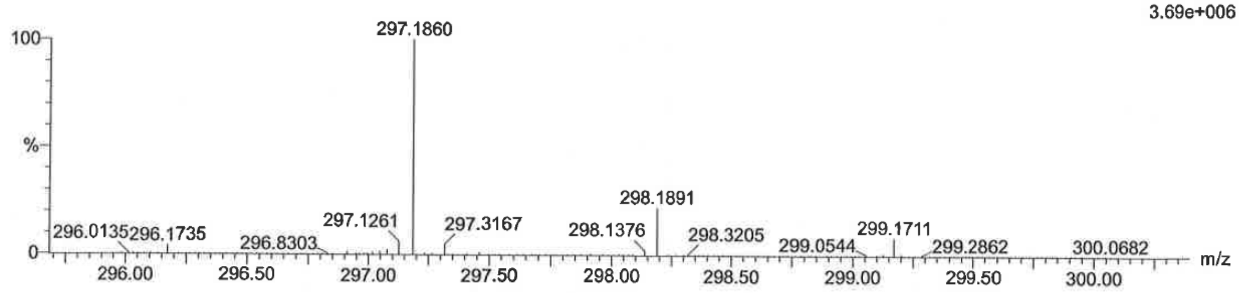

Minimum:				-1.5				
Maximum:		2.0	10.0	80.0				
Mass	Calc. Mass	mDa	PPM	DBE	i-FIT	Norm	Conf(%)	Formula
297.1860	297.1855	0.5	1.7	8.5	1215.5	n/a	n/a	C20 H25 O2

HRMS spectrum of **4** (negative-ion mode)
